# Phosphorus Kβ X-ray emission spectroscopy detects non-covalent interactions of phosphate biomolecules *in situ*†

**DOI:** 10.1039/d1sc01266e

**Published:** 2021-04-29

**Authors:** Zachary Mathe, Olivia McCubbin Stepanic, Sergey Peredkov, Serena DeBeer

**Affiliations:** Max Planck Institute for Chemical Energy Conversion Stiftstr. 34-36 D-45470 Mülheim an der Ruhr Germany serena.debeer@cec.mpg.de

## Abstract

Phosphorus is ubiquitous in biochemistry, being found in the phosphate groups of nucleic acids and the energy-transferring system of adenine nucleotides (*e.g.* ATP). Kβ X-ray emission spectroscopy (XES) of phosphorus has been largely unexplored, with no previous applications to biomolecules. Here, the potential of P Kβ XES to study phosphate-containing biomolecules, including ATP and NADPH, is evaluated, as is the application of the technique to aqueous solution samples. P Kβ spectra offer a detailed picture of phosphate valence electronic structure, reporting on subtle non-covalent effects, such as hydrogen bonding and ionic interactions, that are key to enzymatic catalysis. Spectral features are interpreted using density functional theory (DFT) calculations, and potential applications to the study of biological energy conversion are highlighted.

## Abstract 2

Phosphorus X-ray emission spectroscopy probes non-covalent interactions and electronic structure of phosphate biomolecules in both solid and solution samples.

## Graphical abstract

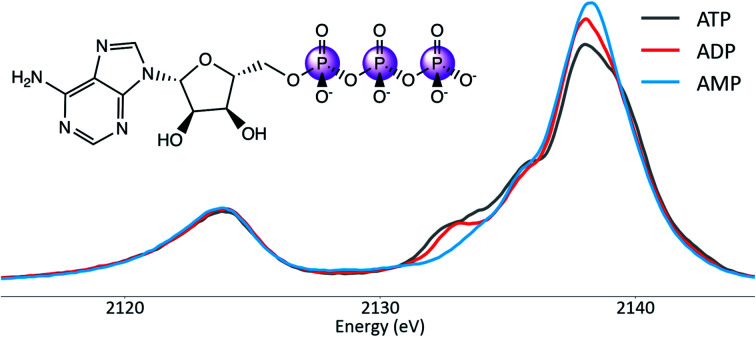

## Introduction

Phosphorus, biologically available in the form of phosphates (species containing the functional group [P^V^O_4_]^3−^), is required for almost all cellular metabolic processes, and nearly 1/3 of known protein structures interact with or contain some form of phosphate.^1,2^ Many central biomolecules, including deoxyribonucleic acid (DNA), ribonucleic acid (RNA), adenosine triphosphate (ATP) and nicotinamide adenine dinucleotide phosphate (NADP), are organophosphates.^3^ The energy transduction reaction of ATP to form ADP (adenosine diphosphate) and orthophosphate (any conjugate base of H_3_PO_4_; “P_i_”) is utilized by a wide variety of proteins to drive otherwise endergonic reactions,^4^ including the reduction of N_2_,^5^ transportation of ions (Na^+^, K^+^, Ca^2+^) across membranes,^6–8^ and formation of NADP.^9^ NADP and NADPH participate in redox reactions in protein environments,^10^ and the mechanism of cisplatin, the original transition metal anticancer drug, involves the mediation of NADPH oxidase.^11^ Phosphates also influence protein folding *via* hydrogen bonds to the basic amino acids and chelates with arginine,^12^ and control conformational gating, such as in the ATP deactivation of ribonucleotide reductase.^13^ Enzyme-inhibiting organophosphates are also the main class of pesticides and nerve agents.^14^ Due to the wide biological importance of phosphates, it is of great interest to follow the chemical changes of phosphate-containing molecules in biological systems.

Phosphate is optically silent in both the visible and ultra-violet regimes. Even in compounds such as NADP and NADPH, which can be differentiated through UV/vis,^15^ the presence of protein in the sample limits the use of this technique due to the large characteristic absorption at 280 nm for all proteins. Although the protonation of orthophosphates can be determined through IR, this technique is less useful for larger compounds such as proteins and is often impossible in aqueous environments, where similar stretches in water obscure relevant transitions.

As such, the majority of phosphorus studies in biological systems have been conducted with ^31^P nuclear magnetic resonance (NMR) spectroscopy, which has been widely applied as a diagnostic tool or for long-range structural characterization.^5,16–24^ Notably, because of the small natural linewidth of NMR, it is useful for the identification of multiple phosphorus species in mixtures. Additionally, ^31^P NMR spectroscopy has been used to investigate structure–activity relationships in organometallic complexes.^16^ There are, however, some drawbacks to NMR spectroscopy. In paramagnetic systems, line broadening and NMR shifts have limited the number of successful studies. Recent studies have utilized *in situ* paramagnetic NMR spectroscopy in characterization of catalysts through optimization of instrumentation.^25,26^ Larger proteins are less amenable to study with solution-state NMR because of strong life-time broadening that occurs with molecular tumbling, limiting the study of such proteins to solid state NMR with sedimented protein samples.^27,28^ Notably, ^31^P NMR inherently has a long relaxation time that is dependent on local environment,^29^ with relaxation times from 0.1 to over 10 seconds.^30^ This property limits the utility of ^31^P NMR spectroscopy in investigating reactivity or conformational changes which, in biochemical systems, typically occur on a scale of milliseconds or less.^31^ As such, there is a need for techniques capable of probing phosphorus sites on faster time scales and in diverse environments, especially in the presence of paramagnetic species and in solution.

X-ray absorption spectroscopy (XAS) is a well-established technique for local structural characterization of both transition metals and main group elements. Sulfur compounds, which often have similar structures to their phosphorus counterparts, give rise to rich features in the XANES region.^32^ X-ray absorption and scattering techniques have been applied to phosphates in both solid and solution phase, albeit with limited success. K-edge XAS of phosphorus suffers somewhat in the EXAFS region because of the generally weak oscillations associated with a low atomic number.^33^ XANES has been used more frequently: a fingerprinting library of various phosphates has been compiled at both the K edge^34^ and the L_2,3_ edge,^35^ and metal–ligand covalency has been studied in phosphine complexes.^36,37^ While XANES spectra of other phosphorus compounds have rich features, the XANES spectra of orthophosphates recorded by Persson *et al.* were described as nondifferentiable due to a lack of distinctive spectral features.^38^ Despite this conclusion, some phosphate salts with transition metal cations offer distinctive pre-edge features,^39^ hinting that P XAS may have more to offer and that probing the valence electronic structure at phosphate can report on the local environment.

In recent years, Kβ X-ray emission spectroscopy (XES) has become a more frequently used technique, with a focus on 3d transition metals.^40–46^ Some work has also been performed on 4d elements,^47–49^ and sulfur Kβ X-ray emission has been somewhat explored,^50–54^ including a study of the protonation state of aqueous sulfate,^55^ but very few Kβ XES studies of phosphorus have been reported.^50,56–58^ Phosphorus Kβ X-ray emission spectroscopy (XES) has great potential as a probe of phosphorus in both molecular and biological systems, promising rich electronic structural information and compatibility with diverse sample preparations that would augment existing techniques. Additionally, while P XAS suffers dramatically from self-absorption,^59^ XES samples are exceptionally easy to prepare and require no dilution. Compared to NMR studies, for which the full experiment can take hours,^56^ or XAS, where sample dilution results in longer collection times, XES of high phosphorus content (for example, Na_4_ATP is 5.8% phosphorus by weight) powder samples requires negligible time to collect (powdered sample collection of Na_4_ATP, as discussed below, took 8 minutes). To our knowledge, P Kβ XES has not been used to study biologically relevant organophosphate compounds or to examine phosphorus compounds in the solution phase. This is in part because of the technical challenges in accessing the energy range required for phosphorus Kβ XES. While there are limited beamlines amenable to P Kβ XES, the ability to perform these experiments using both laboratory and synchrotron instrumentation has been recently demonstrated and applied to simple phosphate salts.^60^ In combination with experimental XES results, density functional theory (DFT) calculations allow for a direct link between the phosphate local environment and the observed spectral features.

To investigate the potential applications of P Kβ XES in a biological setting, the molecules relevant to ATP hydrolysis and NADP redox reactions were studied in both the solid and solution phases. P Kβ spectra were found to be sensitive to subtle non-covalent effects and were correlated to DFT calculations to investigate the origins of spectral features and the influence of ionic interactions and hydrogen bonding. The potential of P Kβ XES for future applications in biochemistry is highlighted.

## Experimental

### Sample preparation

NaH_2_PO_4_, Na_2_HPO_4_, β-nicotinamide adenine dinucleotide phosphate disodium salt (NADP), β-nicotinamide adenine dinucleotide 2′-phosphate reduced tetrasodium salt hydrate (NADPH), 2-amino-2-(hydroxymethyl)-1,3-propanediol (Tris base), adenosine monophosphate (AMP), adenosine diphosphate (ADP), HCl and NaOH were purchased from Sigma Aldrich and used without further purification. Na_2_ATP × 3H_2_O (MP Biomedical) was used for solid samples and Na_2_ATP with 8% H_2_O (Sigma Aldrich) was used for solution samples. Solutions were prepared using ultrapure water (18.2 MΩ cm).

All solid samples were prepared as fine powders in aluminum spacers with 5 μm polypropylene windows. Phosphate solutions were prepared from Na_2_HPO_4_, dissolved to 1.9 M and adjusted to a pH of 9.8 or 4.7, to obtain a single phosphate species (HPO_4_^2−^ or H_2_PO_4_^−^) in solution. NADPH and NADP were dissolved in a pH 8 tris(2-amino-2-(hydroxymethyl)-1,3-propandiol) buffer (Sigma Aldrich). ATP and ADP 30 mM solutions were prepared at a pH of 8 without additional buffer. The purity and stability of ATP and ADP solutions were verified by both ^1^H and ^31^P NMR spectroscopy.

Solutions were measured using a custom flow cell (see ESI Section 1†) machined from polyether ether ketone (PEEK) with an 8 μm Kapton window and a Viton gasket, together with a syringe, syringe driver and standard high-performance liquid chromatography fittings. Constant solution flow provided both dissipation of the significant heat load from the incident beam on the window and replacement of potentially beam-damaged sample with fresh sample. Additionally, the beam was constantly rastered across the window at a speed of 50 μm s^−1^ in the direction opposite of sample flow. The linear flow speed across the window at the point of measurement was approximately 200 μm s^−1^; doubling the speed did not result in any spectral changes, suggesting that beam damage at 200 μm s^−1^ is unlikely. Before collecting data on each sample, the system was thoroughly rinsed with 0.01 M HCl, 0.01 M NaOH, ultrapure water and the solution to be measured.

### XES measurements

All P Kβ XES data were collected at ambient temperature at the PINK beamline at the BESSYII synchrotron.^61^ The incident beam provided by a cryogenically cooled U17 undulator tuned to 4 keV and monochromatized with a multilayer monochromator with a broad bandpass of *E*/Δ*E* ≈ 50. The incident beam had a high flux of 2 × 10^13^ photons s^−1^ and a spot size of 40 × 500 μm (V × H). Emission spectra were collected using a vacuum von Hamos spectrometer with a GreatEyes CCD detector. A Si(111) cylindrical crystal (2*d* = 6.271 Å) with radius of 250 mm was used as a dispersive element. The experimental geometry allowed collection of the emitted photons at a Bragg angle of 69.3–66.7°, corresponding to an energy window of 2110–2150 eV at a CCD resolution of ∼0.036 eV/pixel. The analyzer resolution was estimated to be 0.26 eV (see ESI Section 2†). While the present experiments were performed with the sample chamber under vacuum, with trivial modifications, the sample chamber could be instead filled with 1 atm helium to allow measurement of vacuum-incompatible samples at a cost of ∼2% signal intensity (see ESI Section 1†).

Energy calibration of the spectrometer was performed by fitting the spectrum of a NaH_2_PO_4_ standard with four Voigt peaks, then applying a linear transformation to match literature values of 2139.5, 2137.9, 2135.3, and 2123.4 eV.^58^ NADP and NADPH were characterized using UV/vis spectroscopy before and after beam exposure, and no evidence of beam-induced damage was observed.

### XES data processing

All spectra were normalized with respect to the incident flux and measurement time and linearly interpolated onto a uniform 0.05 eV grid. A blank spectrum of ultrapure water was subtracted from solution samples, then all spectra were baseline-corrected with a line fitted to the regions [2111.35, 2113.35] and [2146.90, 2148.90] and a very mild Whittaker-Eilers smoothing was applied (*λ* = 4).^62,63^ Unless otherwise noted, spectra are presented normalized to a total area of 1000. All data processing was accomplished using in-house python code.

### XES calculations

The ORCA 4.2.0 electronic structure program package from Neese and coworkers was used to perform all calculations.^64^ Cartesian coordinates for all compounds were built with Avogadro,^65^ then geometry optimized using a BP86 functional^66^ with a def2-TZVP basis set^67^ and def2/J auxiliary basis set.^67^ Solvation effects were accounted for using the CPCM water model and a van der Waals Gaussian surface in initial calculations. KDIIS^68^ and SOSCF^69^ were used for improved convergence. XES calculations were performed using an established one-electron ground state DFT protocol, including an absolute energy shift of 69 eV to align with experiment.^40^ Quantitative analysis of ORCA output files was aided by MOAnalyzer.^70^

Spectra were calculated from DFT transitions using a Voigt function with a Lorentzian *Γ* = 0.47 eV and a Gaussian FWHM = 1.55 eV (*σ* = 0.66 eV). While the experimental broadening of the spectrometer was estimated to be significantly smaller (FWHM = 0.61 eV; *σ* = 0.26 eV), this value of *σ* was chosen to approximate the spectral contributions of any multielectron transitions (such as shake or radiative Auger transitions^71–74^), site heterogeneity and other effects not accounted for in the DFT calculations. See Section 1 of the ESI† for further discussion.

## Results and analysis

### Theory of P Kβ XES

**Fig. 1:**
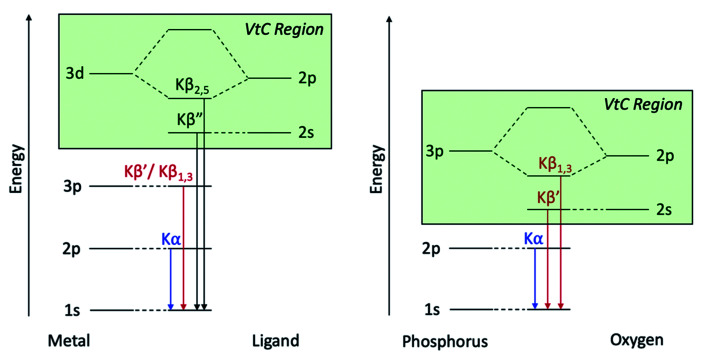
Energy level diagrams for 3d transition metal and ligand binding (left) and for phosphorus and oxygen (right) with the VtC region highlighted in green for both.

To understand Kβ XES of third row main group elements, it is useful to compare with the better-known Kβ lines of first row transition metals. The approximately atomic Kβ mainline (3p → 1 s) of 3d transition metals results in two peaks, Kβ_1,3_ and Kβ′, which report on spin state and metal–ligand covalency. To higher energy, the weaker Kβ_2,5_ and Kβ′′ peaks make up the valence-to-core (VtC) XES region and serve as a probe of the filled valence molecular orbitals (MOs), which provide information on ligand identity, electronic structure and protonation state.^75–77^ VtC transitions derive from MOs of primarily ligand *n*s/*n*p character (Fig. 1, left), with transition intensity resulting from a small amount of metal p character mixed into the ligand orbitals. Contributions from the filled 3d orbitals may also be observed, provided there is sufficient metal p character in these MOs.^78–80^

Third-row main group elements differ from first-row transition metals in that their valence orbitals, those most capable of mixing with ligand orbitals, are 3p rather than 3d. Thus, phosphorus Kβ mainline transitions are not approximately atomic, but rather reflect the valence bonding environment analogous to transition metal VtC transitions while being more strongly dipole-allowed. Comparative energy level diagrams in Fig. 1 illustrate these transitions as they correspond to the various regions of the X-ray emission spectrum.

In P Kβ XES, spectral intensity from a given transition is due to the dipole allowedness of said transition. For a transition to be dipole-allowed, the integral of the product of the dipole operator with the initial and final states must be nonzero:〈final|dipole|initial〉 ≠ 0

Or, in a one-electron approximation, the integral of the product of the dipole operator with the initial and final orbitals must be nonzero:〈1s|dipole|valence〉 ≠ 0

Symmetry can be used to determine when this integral may be nonzero, and thus when the corresponding transition may have spectral intensity. The 1s orbital (|1s〉) transforms as the totally symmetric representation because it is approximately spherical:*Γ*^(1s)^ = *Γ*^(tot-symm)^

Thus, in order for the (one-electron) dipole integral to be nonzero, the direct product of the irreducible representations of the dipole operator and valence orbital must contain the totally symmetric representation:*Γ*^(dipole)^ ⊗ *Γ*^(valence)^ ⊃ *Γ*^(tot-symm)^

This equation is true if and only if the dipole operator and the valence orbital transform as the same irreducible representation, giving the following general requirement for allowed P Kβ transitions:*Γ*^(dipole)^ = *Γ*^(valence)^

This requirement is independent of the point group of the species under consideration. It should be noted that this requirement is a necessary but not sufficient condition for a transition to have nontrivial intensity. A transition may be symmetry-allowed yet have vanishing intensity due to *e.g.* low orbital overlap.

**Fig. 2:**
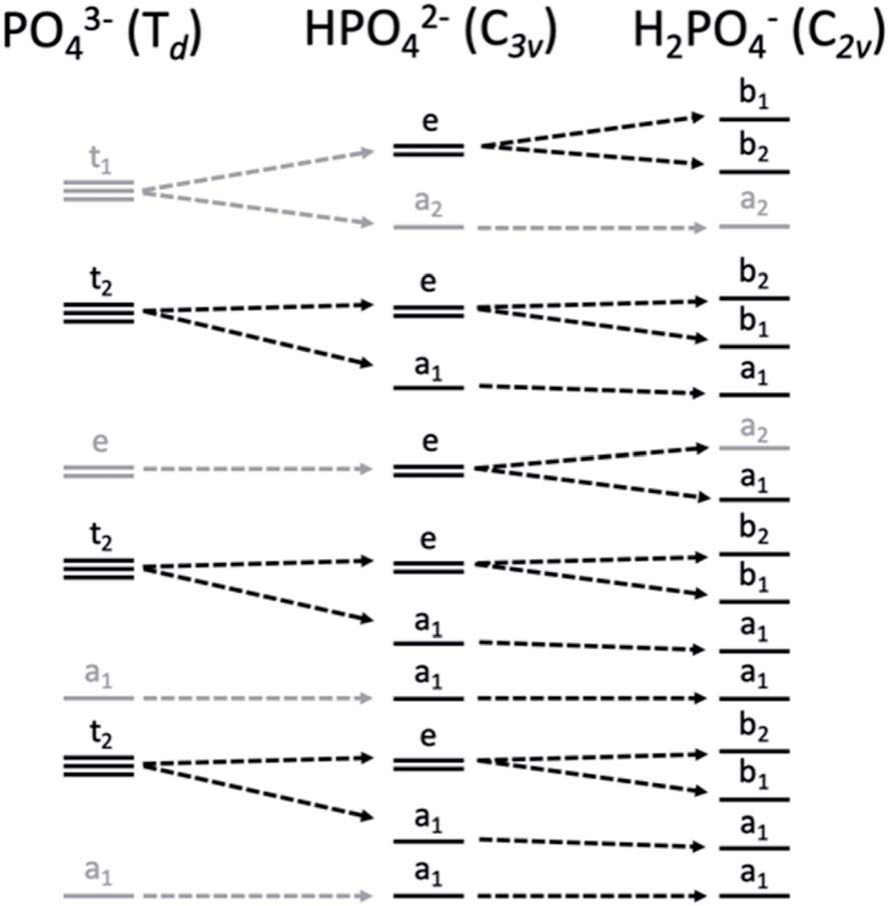
Correlation diagram of orbital symmetries in the *T*_d_, *C*_3v_ and *C*_2v_ point groups, with those matching the symmetry of the dipole operator of each point group in black and others in gray.

Within the orthophosphate series, PO_4_^3−^, HPO_4_^2−^ and H_2_PO_4_^−^, the approximate symmetry decreases with protonation, from *T*_d_ to *C*_3v_ to *C*_2v_, respectively. Within *T*_d_ symmetry, the dipole operator transforms as t_2_, and thus the valence orbital must also transform as t_2_ for the transition to be dipole-allowed. As the symmetry is lowered by protonation, t_2_-symmetric functions (orbitals and operators) transform instead as e and a_1_, then a_1_, b_1_ and b_2_ (Fig. 2). The total number of allowed transitions increases with symmetry reduction because more orbitals transform as the same representation as a dipole operator component.

**Fig. 3:**
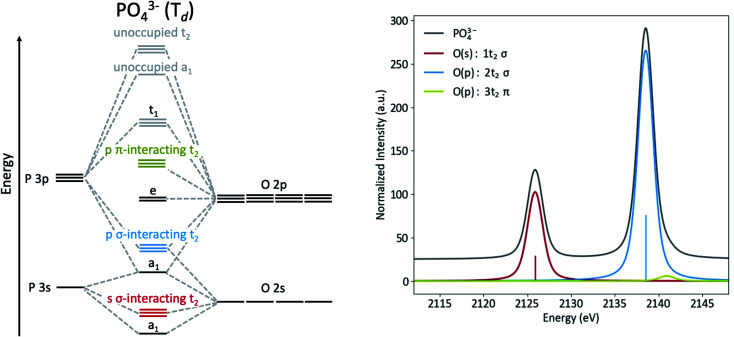
MO diagram (left) and calculated Kβ spectrum (right) of PO_4_^3−^.

The origins of the MOs of PO_4_^3−^ are represented by the molecular orbital diagram shown in Fig. 3. Here, the three sets of t_2_ orbitals can be correlated to three peaks in the calculated Kβ spectrum. The colored molecular orbitals shown in Fig. 3 (left) correspond to the similarly colored peaks in the spectrum on the right. As previously reported by Petric and coworkers (and as calculated in Fig. 3, right), the Kβ spectrum of PO_4_^3−^ has two main peaks that correspond to orbitals with O(2s) and O(2p) character, as well as a small shoulder to high energy that corresponds to orbitals with O(2p) character. The lowest energy peak (red) at 2126 eV arises from t_2_ molecular orbitals with significant O(2s) character. The most intense peak (blue, 2138.5 eV) and weak shoulder towards higher energy (green, 2141 eV) both originate from t_2_ molecular orbitals with primarily O(2p) character. The difference in intensity for these peaks can be explained by the orbital overlaps. The high-intensity blue peak is dominated by σ-interactions of the P(3p) and O(2p) orbitals, while the low intensity green shoulder is due to π-interacting O(2p) orbitals.

**Fig. 4:**
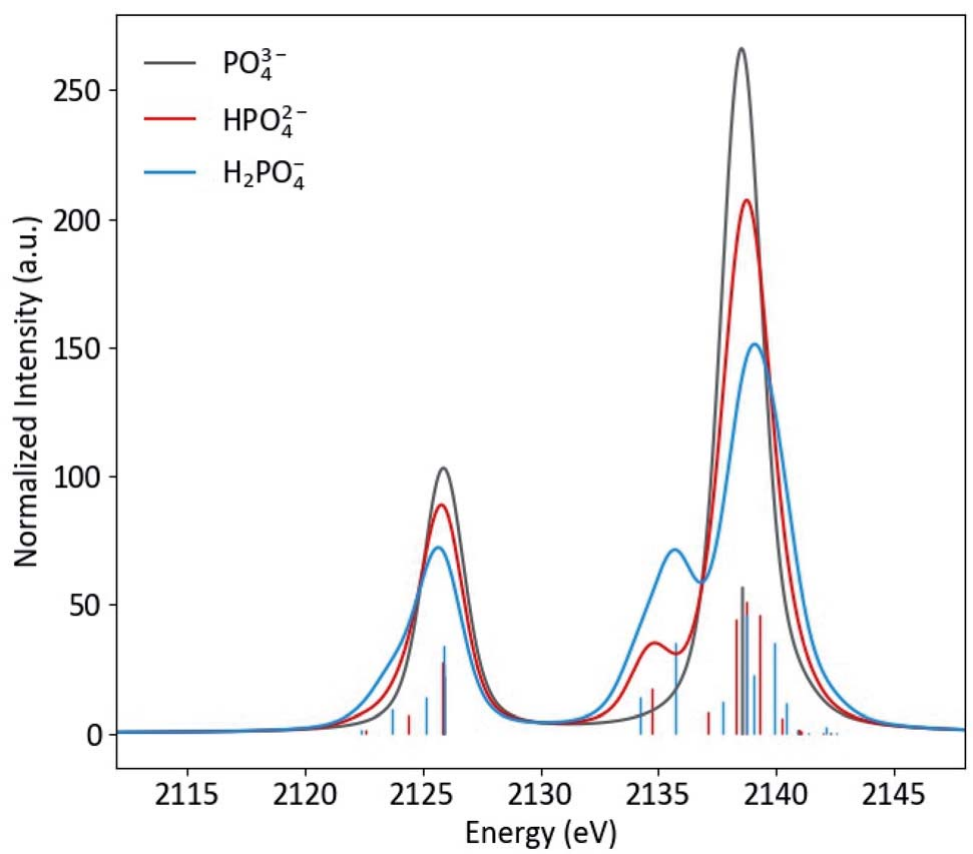
Calculated Kβ spectra of PO_4_^3−^, HPO_4_^3−^ and H_2_PO_4_^3−^ with individual transitions shown as sticks.

Protonation to HPO_4_^2−^, with the subsequent decrease in symmetry and change in orbitals that transform as the same representation as the dipole operator (left to right, Fig. 2), results in a spectra notably different from PO_4_^3−^, with the most pronounced change being the presence of a new peak at 2135 eV (Fig. 4). This peak corresponds to the a_1_ orbital that contains O(2p) character from the protonated oxygen. The peak at 2138.5 eV has contributions from one a_1_ and two e orbitals, which are close enough in energy to appear as a single peak. The spectral change from PO_4_^3−^ to HPO_4_^2−^ is mimicked at lower energy, where a small shoulder at 2124 eV is present, due to the a_1_ orbital containing O(2s) character from the protonated oxygen.

As is shown in Fig. 2, all orbital degeneracy is lost upon protonation to H_2_PO_4_^−^. This results in a somewhat broader peak at 2138.5 eV. With two protonated oxygens, the peak at 2135 eV increases dramatically, as is mimicked at lower energy with the small shoulder at 2123 eV. An overestimation of the MO splitting due to oxygen protonation in the DFT calculations has been observed previously in studies of Ca and Mn VtC XES.^41,77^ As such, it is likely that the peak broadening that occurs in calculated spectra will be somewhat less apparent in experimental spectra.

### XES of phosphate in solution

**Fig. 5:**
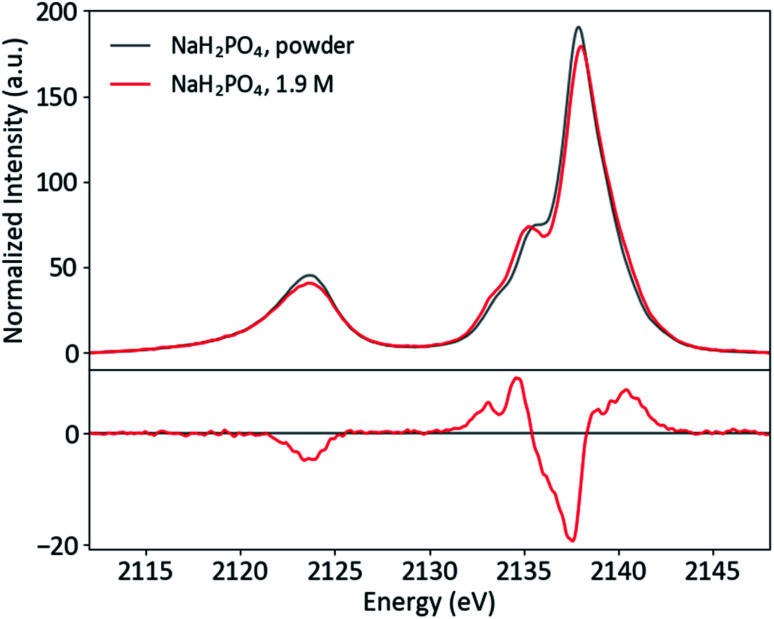
Powder and solution P Kβ spectra of NaH_2_PO_4_, with difference (solution–powder).

Though P Kβ XES of phosphate salts has been discussed in previous publications,^57,58^ phosphate biochemistry occurs in solution and XES of concentrated solid samples is of limited practical use for biochemical applications. To further XES as a means to study biological phosphates, solution samples of the orthophosphates HPO_4_^2−^ and H_2_PO_4_^−^ were prepared. The pH was adjusted to 4.7 and 9.8 for H_2_PO_4_^2−^ and HPO_4_^2−^, respectively, resulting in solutions with 99% homogeneity of protonation state. Both solutions were made with a concentration safely below the maximum solubility (1.9 M for H_2_PO_4_^2−^ and 0.2 M for HPO_4_^2−^). Solid and solution P Kβ spectra of NaH_2_PO_4_ and Na_2_HPO_4_ are presented in Fig. 5 and ESI Section 3,† respectively, with differences (solution–powder) to highlight effects of the phase. Between the two species, the major difference occurs in the Kβ mainline region from 2133 to 2145 eV, with the second protonation resulting in additional spectral features and higher intensity around 2135 eV.

A comparison of the solid and solution spectra of NaH_2_PO_4_ illustrates that P Kβ XES is sensitive to non-covalent interactions, such as hydrogen bonding and intermolecular electrostatic effects, beyond the orthophosphate molecule. While the changes are subtle, it is clear that the shoulder at ∼2136 eV becomes more pronounced in the solution case. Here it is of interest to first discuss what is known about the solid and solution structures of NaH_2_PO_4_, in order to understand the origins of the observed spectral differences and motivate a brief computational study.

Detailed structural information is available for H_2_PO_4_^−^, most notably from a neutron diffraction crystal structure of NaH_2_PO_4_ and from various spectroscopic and computational studies in solution. The NaH_2_PO_4_ crystal structure contains two unique H_2_PO_4_^−^ sites in the asymmetric unit that are, on average, acceptors in 6Na^+^ and 2HO–PO_3_H interactions and donors in 2O–PO_3_H_2_ interactions, with an average intermolecular O–O distance of 2.56 Å.^81^ In solution, the hydration structure of H_2_PO_4_^−^ is not precisely known; in general, it is difficult to establish agreement in hydration metrics across different techniques for such ions (see Eiberweiser *et al.* 2015 for further discussion).^82–86^ There is, however, substantial experimental and computational agreement that H_2_PO_4_^−^ is strongly hydrated, with a first solvation sphere containing at least enough water molecules to saturate the hydrogen bonding sites in a static Lewis picture (9 hydrogen bond acceptances and 2 donations). The average intermolecular O–O distance is longer than that in the crystal, calculated at 2.77 and 2.79 Å from large-angle X-ray scattering and infrared spectroscopic data, respectively.^82,83^ The fastest hydration dynamics at orthophosphate occur on a timescale >10^−12^ s, much slower than the P(1s) core hole lifetime of 10^−15^ s; thus P XES reflects a sum across solution species, analogous to the slow-exchange regime in NMR.^84,86,87^

Overall, H_2_PO_4_^−^ has a similar number of non-covalent interactions in both environments, with 6 Na^+^ interactions and 4 short hydrogen bonds in the solid and ∼11 hydrogen bonds that are ∼0.2 Å longer in the solution. Compared to the largely electrostatic nature of Na^+^ interactions, hydrogen bonding is a complex phenomenon with contributions from electrostatic, covalent (charge-transfer) and dispersion (intermolecular electron correlation) interactions that can alter the electronic structure of the acceptor moiety.^88–91^

**Fig. 6:**
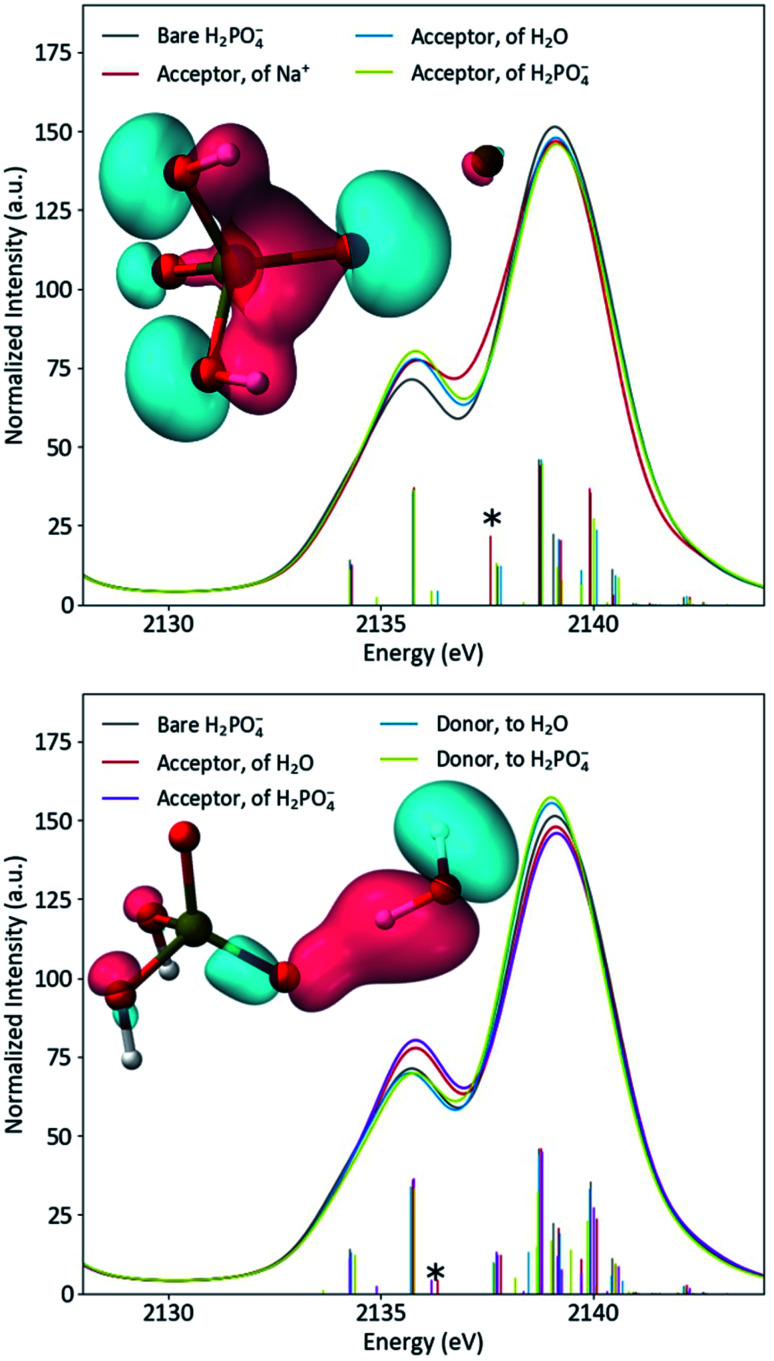
Calculated spectra of bare H_2_PO_4_^−^ compared to acceptor dimers (top) and hydrogen bonding dimers (bottom), with inset plots of the starred transitions' orbitals.

The differing effects of the two coordination environments on the H_2_PO_4_^−^ Kβ_1,3_ region were explored briefly in a series of single-point DFT calculations on continuum-solvated optimized dimer structures (Fig. 6). One might guess that the increased width of the solution Kβ_1,3_ region simply implies a stronger orbital splitting, analogous to that observed upon symmetry reduction (*vide supra*). However, the calculations suggest that the smoother shoulder in the solid spectrum is a result of stabilization of an orbital of primarily O(2p) character due to the proximal Na^+^. Despite the electrostatic, non-bonding nature of the interaction and the near-equivalence of the plotted MOs, the transition probability for this orbital is increased by 77% in the presence of Na^+^. In dimers with either water or another H_2_PO_4_^−^, the spectrum is most affected when the probed H_2_PO_4_^−^ is the acceptor of the hydrogen bond, as expected. In these spectra, a new covalent hydrogen bonding orbital (that lacks a clear correlate in the monomer) contributes significant shoulder intensity. The spectra do not depend significantly on whether the hydrogen bonding partner is water or orthophosphate. It is clear that, in real systems, DFT is a powerful tool for understanding P Kβ spectra. A more thorough DFT study of these effects, employing the full crystal structure and explicit-solvent molecular mechanics, is underway in our group.

### Adenine nucleotides

**Fig. 7:**
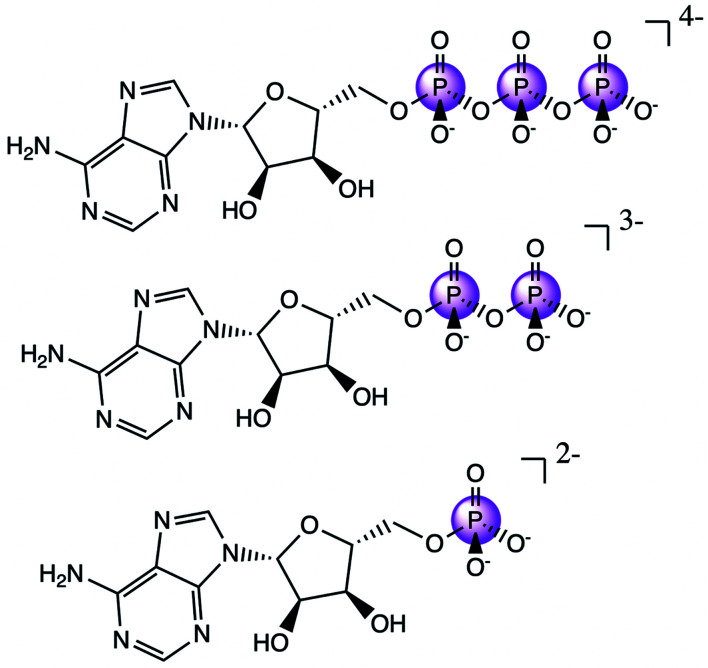
Molecular models of ATP (top), ADP (middle), and AMP (bottom).

**Fig. 8:**
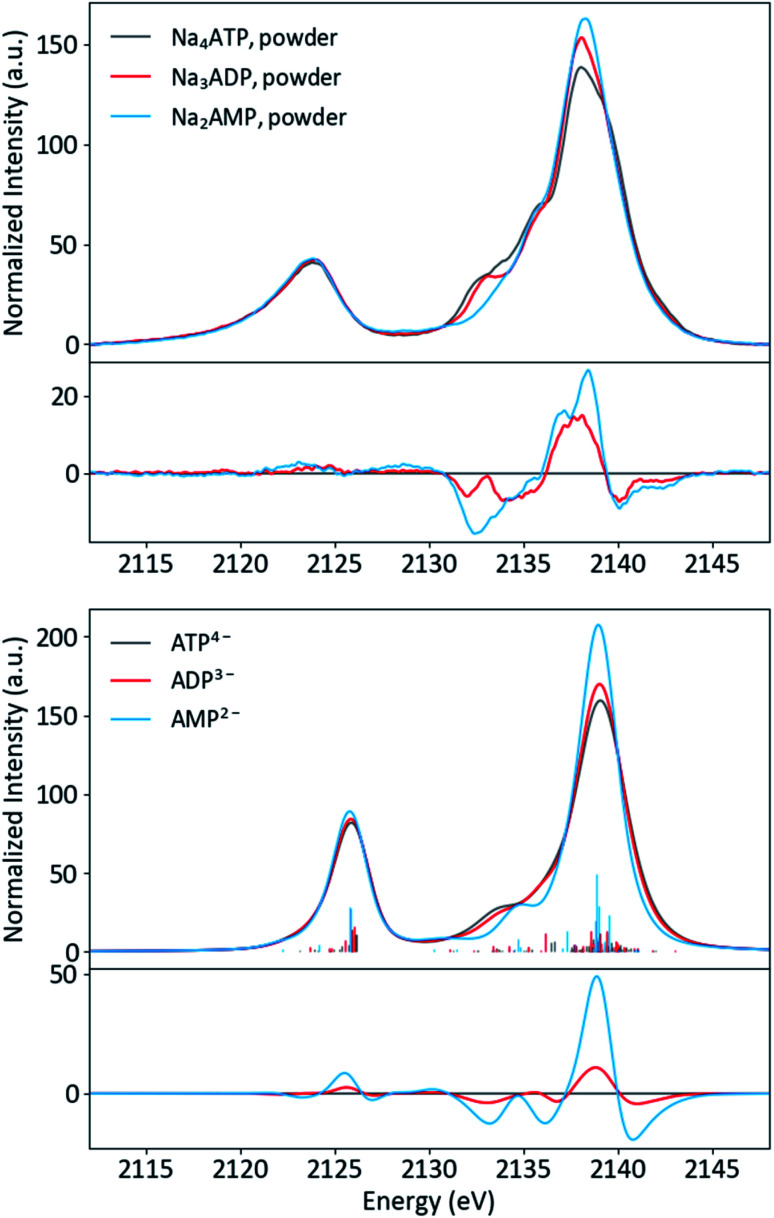
Experimental (top) and calculated (bottom) spectra of ATP, ADP and AMP salts, with differences (AXP–ATP).

Having an understanding of the MO structure of P_i_, we now move to more complex phosphate-containing biomolecules. The changes in the P Kβ XES with phosphate bound to another phosphorus, as occurs within ATP and ADP, have not, to our knowledge, previously been reported. As such, it is useful to analyze the adenine nucleotide series (AXP, where X= mono- (M), di- (D) or tri- (T)), the molecular models of which are shown in Fig. 7. The experimental spectra of these three complexes are shown in Fig. 8 together with calculations. As found with the orthophosphate series, Kβ′ peaks at 2123 eV do not vary strongly between species. The peaks in the Kβ_1,3_ region, derived from orbitals with O(p) character, decrease in intensity at 2132 eV and increase in intensity 2138 eV with a decreasing number of phosphates. The calculations match well overall with experiment, and the corresponding trends are clear in the difference spectra. Calculations confirm that P Kβ spectra of these compounds report on the phosphate local environment in particular, with the ribonucleotide having a negligible influence (see ESI Section 4†). The differences between ADP and ATP in particular are rather small, but the change in intensity at 2130 eV and the broadening of the peak at 2139 eV for ATP allow for distinction between low-noise spectra.

**Fig. 9:**
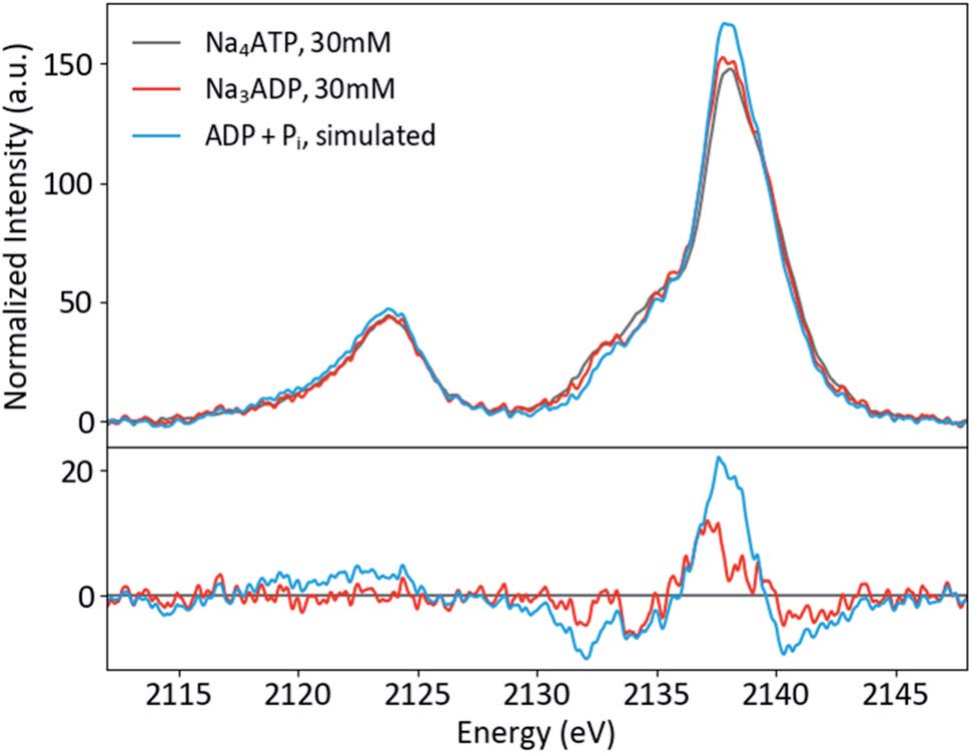
P Kβ spectra of ATP and ADP in solution, plus a simulated spectrum of the reaction product ADP + P_i_, with differences (x-ATP).

Solution phase spectra of ATP and ADP at 30 mM are shown in Fig. 9. As with the powder samples, the spectra of ATP and ADP are distinguishable but not grossly different. However, it is important to consider the biologically relevant reaction that occurs: ATP → ADP + P_i_. As XES is an element-selective probe, the orthophosphate would also contribute to the overall spectra. As such, the most relevant spectral comparison is between ATP and ADP + P_i_, for which the differences become more apparent. In order to correctly approximate this comparison, the pH of the environment needs to be considered. A mixture of species (13% H_2_PO_4_^−^ and 87% HPO_4_^2−^) is present at pH 8, so the weighted average of two single-species spectra was used to create a pH 8 P_i_ spectrum. The weighted 1 : 2 pH 8 P_i_ : ADP spectrum shown in Fig. 9 properly accounts for the change that would occur to the P XES spectra upon ATP hydrolysis. An increase in peak intensity at 2138 eV and a decrease in the shoulder features between 2130 and 2135 eV are clearly visible in the difference spectrum *versus* ATP. The ATP spectrum also appears somewhat broader than that of its mixed spectrum counterpart.

Adenine nucleotide speciation in solution is complex and dependent on the concentration, counterions and pH, and the conditions here (30 mM and pH 8) were chosen to minimize the distribution of species while maintaining a practical signal intensity for commissioning-type experiments. With increased concentration and in the presence of Mg^2+^ (which forms chelate complexes with polyphosphate groups), adenine nucleotides form stacked multimers.^92^ On the basis of known dissociation constants, our solutions were dominated by monomers of ATP^4−^ (90%), MgATP^2−^ (80%), ADP^3−^ (90%) and AMP^2−^ (85%), with the remainders dimerized. MgATP^2−^ exists in two conformational classes, an open form (∼80%) in which Mg^2+^ is ligated only by the phosphate groups and water, and a closed form (∼20%) additionally including adenine ligation.^92,93^ Na^+^ does not bind significantly to ATP^4−^ in solution. In almost all biochemical reactions in which they participate, adenine nucleotides are found in the form of metal ion complexes, typically with Mg^2+^.^92^

**Fig. 10:**
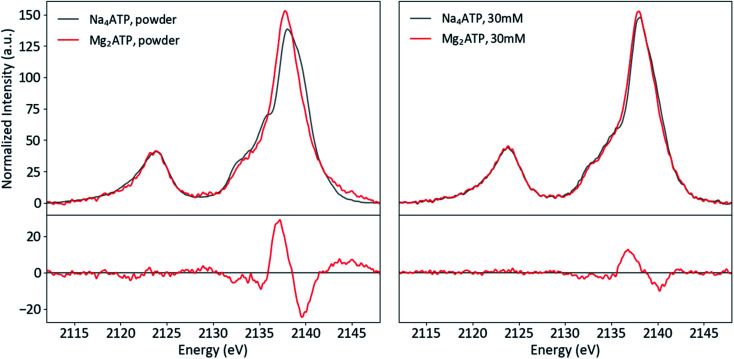
Powder (left) and solution (right) P Kβ spectra of Na_4_ATP and Mg_2_ATP, with differences (Mg_2_ATP–Na_4_ATP).

To investigate the sensitivity of P Kβ XES to cation coordination, spectra of Na_4_ATP and Mg_2_ATP were recorded from powders and solutions (Fig. 10). Parallel differences are visible in both phases: with Mg^2+^, the Kβ_1,3_ peak is sharper, has a less prominent first shoulder at 2135 eV and is shifted to lower energy overall, with peak maxima shifted by 0.2 eV in the powder and by 0.1 eV in solution. The similarity of these changes, made clear in the difference spectra, suggests that the influence of Mg^2+^ on the polyphosphate electronic structure is conserved in both phases. Calculations using free and Mg^2+^-bound structures of ATP with and without Mg^2+^ suggest the dication's influence on the triphosphate conformation and interactions with phosphate orbitals both influence the P Kβ spectra (see ESI Section 5†).

### NADP^+^ and NADPH

**Fig. 11:**
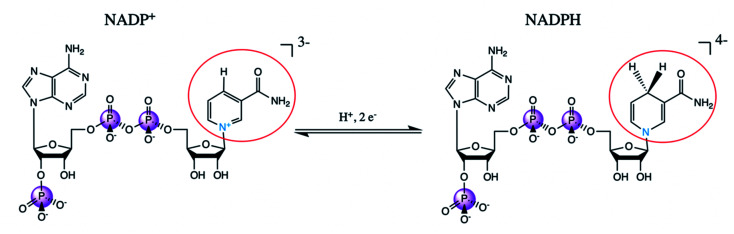
Schematic reaction diagram of NADP^+^ and NADPH, with redox-relevant group circled in red and participating nitrogen shown in blue.

NADP^+^/NADPH is an important biological redox system, with NADPH providing the reducing equivalents for many metabolic reactions. The reduction from NADP to NADPH does not occur at one of the phosphates; rather, a proton and two electrons are added the nicotinamide, removing its aromaticity and formal charge as is depicted in Fig. 11. These distal differences will not influence the phosphorus Kβ spectrum directly through the covalent bonding structure; any spectral differences are due to concomitant conformational changes at the phosphates or intra-/intermolecular interactions with the phosphates. We also note that the NADP^+^ powder is once protonated on one of the phosphates, while NADPH was purchased in its quad-anionic form.

**Fig. 12:**
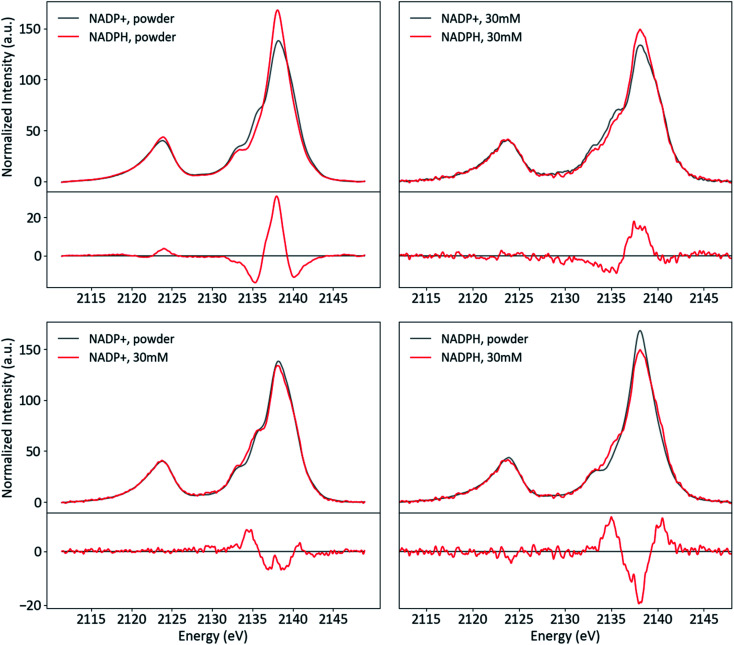
Powder and solution spectra of NADP^+^ and NADPH paired by phase (top) and redox state (bottom), with differences (red-black).

Powder and solution spectra of NADP^+^ and NADPH are presented in Fig. 12, paired to accentuate trends in redox states (top row) and phases (bottom row). The two species differ strongly in the Kβ_1,3_ region, with intensity shifted from the shoulder at 2135 eV to the mainline upon reduction. This change is very similar in both phases, which indicates it is not a result of the powder protonation state. Rather, it appears that the redox state of the nicotinamide influences specific phosphate interactions in a manner that is consistent across phases.

For both species, the shoulder at 2135 eV is more pronounced in solution, while the intensity shift from the mainline (2138 eV) to the high-energy shoulder (2140 eV) is much stronger for NADPH than NADP. The former conserved change, similar to that observed for NaH_2_PO_4_ (*vide supra*), likely results from increased hydrogen bonding and reduced ionic interactions in solution. The latter change may be related to the difference between species that is conserved across phases.

To our knowledge, there is not sufficient published structural information about NADP^+^/NADPH in solid or solution phases to determine the intra- or intermolecular interactions responsible for the observed changes. DFT geometry optimizations of both species typically converged to structures with a hydrogen bond donation from the nicotinamide to the phosphate with which it shares a ribose (see ESI Section 6†). Since reduction changes the aromaticity and formal charge of the nicotinamide (and hence its hydrogen bonding properties), this intramolecular interaction is a reasonable candidate for the mediator between nicotinamide redox state and phosphate electronic structure. In particular, a hydrogen bond that is present in both phases only in NADP^+^ could explain the observations: the higher intensity shoulder at 2135 eV in NADP^+^ could be due to a strong hydrogen bond that persists in both phases, while the lack of such a bond could explain the stronger phase effect at 2038–2040 eV in NADPH. Experimental structural data or a more in-depth computational study are needed to test the above hypotheses or facilitate further speculation.

## Discussion and conclusions

In this study, we have presented the first solution-phase Kβ XES of phosphate biomolecules, demonstrating the feasibility of the technique as well as its impressive sensitivity to non-covalent interactions of broad interest in biochemistry.

X-ray spectroscopy of lighter elements (Z ≲ 22) presents practical challenges, including beam attenuation, sample damage and 1s core-hole fluorescence yield that are unfavorable compared to those of heavier elements. The present experiments benefitted from two redeeming factors: the very high incident flux of the PINK beamline and the high dipole allowedness of P 3p → 1s transitions compared to that of transition metal VtC transitions.^94^ For reader calibration, the solution spectrum of 30 mM Na_4_ATP presented above required 63 minutes of beam exposure time in 156 minutes of clock time, while the corresponding powder spectrum took 8 minutes to collect. We note, too, that all data presented here were from unoptimized experiments conducted during the commissioning of the PINK beamline; with our next-generation sample cell, the clock time could be reduced to approximately the exposure time and the sensitivity improved. Compared to P XAS, sample preparation for P XES is simple and adaptable. With its rich electronic structural information content and rapid solid-state measurement times, P XES will complement the speciation capability provided by the narrower linewidths of ^31^P NMR.

In the orthophosphate series, we used molecular symmetry to obtain a basic understanding of P Kβ X-ray emission. Spectra of H_2_PO_4_^−^ revealed that Kβ_1,3_ features (and hence phosphate valence MOs) are sensitive to the ionic and hydrogen bonding interactions present in solid NaH_2_PO_4_ and its solution. Prior structural data and DFT calculations allowed further assignment of the transitions and emphasized the necessity of a quantum chemical approach to understanding such interactions. Though simple single-point DFT calculations are illuminating, biochemical applications of P Kβ XES would benefit from a complimentary computational approach that samples a large conformational space, such as *ab initio* molecular dynamics or hybrid quantum mechanics/molecular mechanics.^84,95^

Solution P Kβ XES of adenine nucleotides and the NADP^+^/NADPH redox system further demonstrated the ability of the technique to probe reactions and non-covalent interactions in a biochemical context. Monitoring the hydrolysis of ATP to ADP and P_i_ in solution is clearly plausible. Moreover, sensitivity to the conformational and electronic structural consequences of Mg^2+^ binding to ATP and even distal changes like the reduction of NADP to NADPH offers great promise for future studies. Both phosphate-transferring systems and the many enzymatic reactions that rely on phosphate cofactors could be targeted. The specific utility of XES, including time resolution and the potential for multi-modal or two-color experiments is also noteworthy. Beyond biochemistry, *in situ* P Kβ XES could be of particular interest for homogeneous catalysis research: nucleophilic phosphine catalysts are increasingly used to prepare pharmaceuticals, natural products and materials, and phosphorus-based ligands are perhaps the most important class of spectator ligands in transition metal catalysis.^96–99^ Interest in the use of phosphorus in battery electrodes and other advanced materials is also growing.^100–105^ Overall, we believe P Kβ XES could find broad use and impact in fields relevant to chemical energy conversion.

## Author contributions

ZM, OMS and SD conceptualized the project. ZM, OMS and SP developed the methodology and conducted the investigations. ZM and SP curated data and worked on software components, and ZM performed the formal analysis. ZM and OMS validated experiments and visualized data. SD served as the project supervisor. OMS wrote the original draft, and all authors reviewed and edited the manuscript.

## Conflicts of interest

The authors have no conflicts of interest to declare.

## Supplementary Material

SC-012-D1SC01266E-s001
