# Supplementary material for: Phosphorus Kβ X-ray emission spectroscopy detects non-covalent interactions of phosphate biomolecules in situ
Source: Chem Sci. 2021 Apr 29;12(22):7888–901. doi: 10.1039/d1sc01266e (PMC8188515; doi:10.1039/d1sc01266e)
Supplement: SC-012-D1SC01266E-s001 [file SC-012-D1SC01266E-s001.pdf]

Electronic Supplementary Material (ESI) for Chemical Science.
This journal is © The Royal Society of Chemistry 2021

Supporting Information:
Phosphorus Kβ X-Ray emission spectroscopy detects non-covalent interactions of phosphate biomolecules *in situ*

Zachary Mathe,[a†] Olivia McCubbin Stepanic,[a†] Sergey Peredkov,[a] Serena DeBeer[a*]

[a] Max Planck Institute for Chemical Energy Conversion, Stiftstr. 34-36, D-45470 Mülheim an der Ruhr, Germany

† These authors contributed equally

Contents

1. The PINK X-ray Emission Spectrometer and Liquid Cell 2
2. Line Broadening in XES 3
3. Spectra of Na_2HPO_4 4
4. ATP and Ethyl-triphosphate calculated spectra 5
5. MgATP Structures 6
6. NADP and NADPH Structures 7

References 8

1. The PINK X-ray Emission Spectrometer and Liquid Cell

The present experiments were performed at the PINK end station at the BESSY II synchrotron in Berlin. The end station was configured with a sample environment chamber (SEC) and a vacuum von Hamos spectrometer, separated by an 8-µm Kapton film window. The SEC contains a motorized sample stage to which multiple sample cells of various types can be mounted. Gas-tight liquid passthrough into and out of the SEC was achieved with standard HPLC fittings.

Due to the significant attenuation of 2 keV photons by air, the SEC and spectrometer were kept under vacuum at 5 mbar and 10^{-5} mbar, respectively. With minor modifications, the SEC could be filled with one atmosphere of helium instead of vacuum, allowing for study of vacuum-incompatible samples at a cost of ~2% of detection efficiency.

An enlarged diagram of the liquid cell is presented in Figure S1. The flow in/out ports, shown as black circles in the back plate, are recessed in an oval flow channel routed 1 mm deep into the back plate. Two quarter inch ports machined into the reverse side of the back plate allow connection of the liquid cell to standard HPLC components. The liquid cell is held together with four bolts.

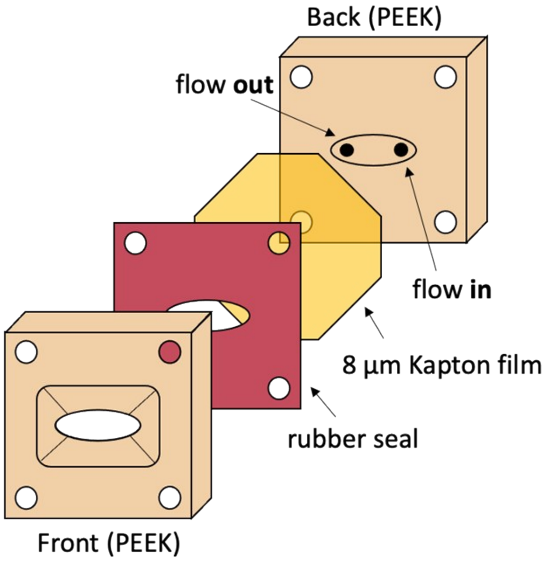

Figure S1. Enlarged view of the liquid sample cell.

2. Line Broadening in XES

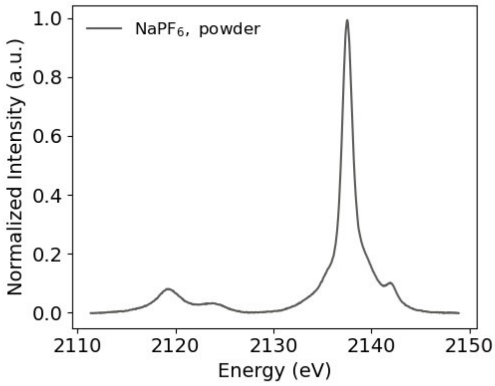

Figure S2. Kβ X-ray emission spectrum of $NaPF_6$

P Kβ X-ray emission spectra are a sum of peaks, each with a Voigt profile. A Voigt function is a convolution of a Lorentzian and a Gaussian function, with respective broadening parameters of Γ and σ. The Gaussian broadening is due to the normal distribution of the spectrometer response function, while the Lorentzian broadening is due to the natural linewidth of the X-ray emission process. In a nonresonant X-ray emission experiment, the observed natural linewidth is the sum of the natural linewidths of the core-hole intermediate state and the valence-hole final state[1,2]:

$$\Gamma_{XES} = \Gamma_{P(1s)} + \Gamma_{valence}$$

Campbell & Papp recommend a value of $\Gamma_{P(1s)} = 0.47$ eV, corresponding to a lifetime of $\tau = 1.4 \times 10^{-15}$ s.[3] This value provides a lower bound for the Lorentzian broadening in non-resonant P K-edge XES in the limit of a long-lived final state.

The sharpest spectrum collected in the present study at the PINK beamline was that of $NaPF_6$ (Figure S2). To investigate the broadening parameters, the $K\beta_{1,3}$ spectrum was fit to a sum of 4 or 5 Voigt functions constrained to have $\Gamma \geq 0.47$ eV (Figure S3). For the main peak, broadenings of $\Gamma = 0.47$ eV and $\sigma = 0.26$–0.27 eV (Gaussian FWHM = 0.61–0.64 eV) were consistently obtained. These values may be taken as upper bounds; thus $\sigma_{spectrometer} \leq 0.26$ eV and $\Gamma_{XES} = 0.47$ eV.

Calculated spectra in this study are presented with broadenings of $\Gamma = 0.47$ eV and $\sigma = 0.66$ eV (Gaussian FWHM = 1.55 eV), with the increased value of σ chosen to obtain a better match with experimental phosphate spectra and to avoid visual clutter. The fact that a larger broadening is required to obtain good agreement between theory and experiment suggests that effects not accounted for in the DFT calculations (such as multi-electron transitions, which can be significant in the Kβ spectra of other elements[4–7]) contribute to P Kβ emission.

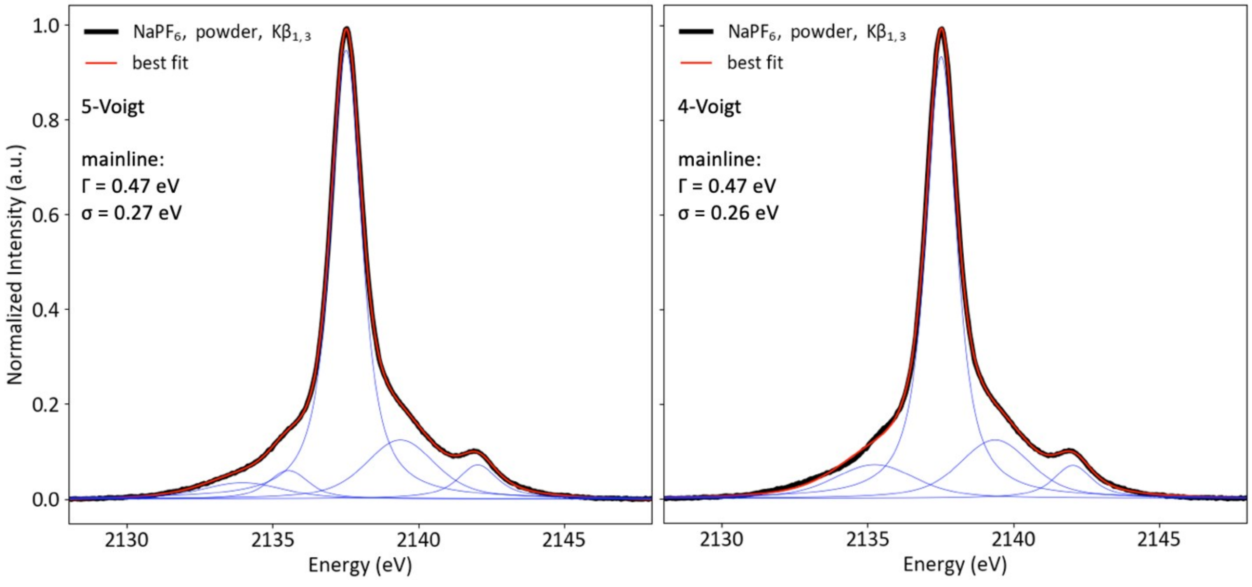

Figure S3. Voigt fits of the $NaPF_6$ $K\beta_{1,3}$ spectrum, with the broadening parameters of the most intense peak inset

3. Spectra of Na_2HPO_4

Powder and solution P Kβ spectra of Na_2HPO_4 are presented in Figure S4. The periodically-oscillating background in the spectrum was a result of technical difficulties with the solution cell during data collection and does not reflect a general limitation of the apparatus.

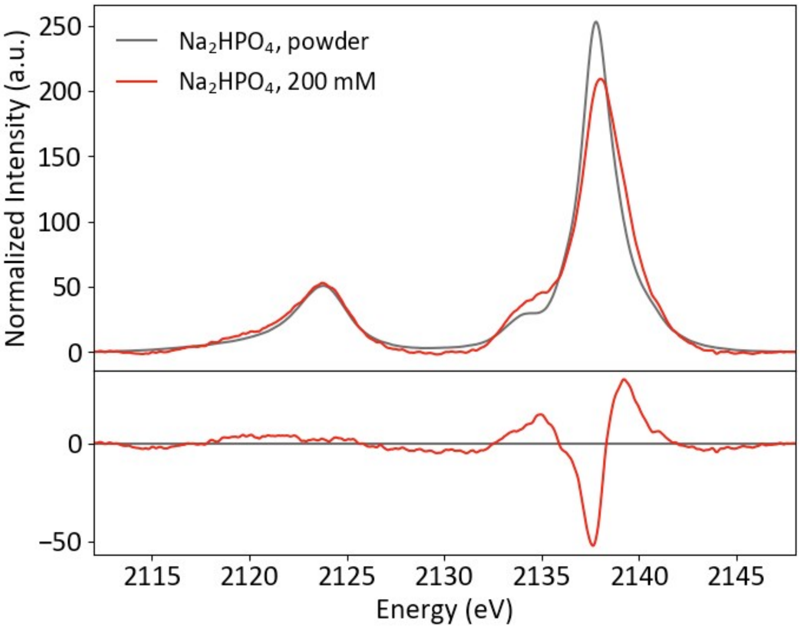

Figure S4. Powder and solution P Kβ spectra of Na_2HPO_4, with difference (solution - powder)

4. ATP and Ethyl-triphosphate calculated spectra

P Kβ spectra are specifically sensitive to the local phosphate electronic structure, not that of whole molecule. To demonstrate the insensitivity of P XES to distal effects, equivalent computed spectra of ATP and ethyl triphosphate, in which the ribonucleotide is replaced with -CH_2CH_3, are presented in Figure S5.

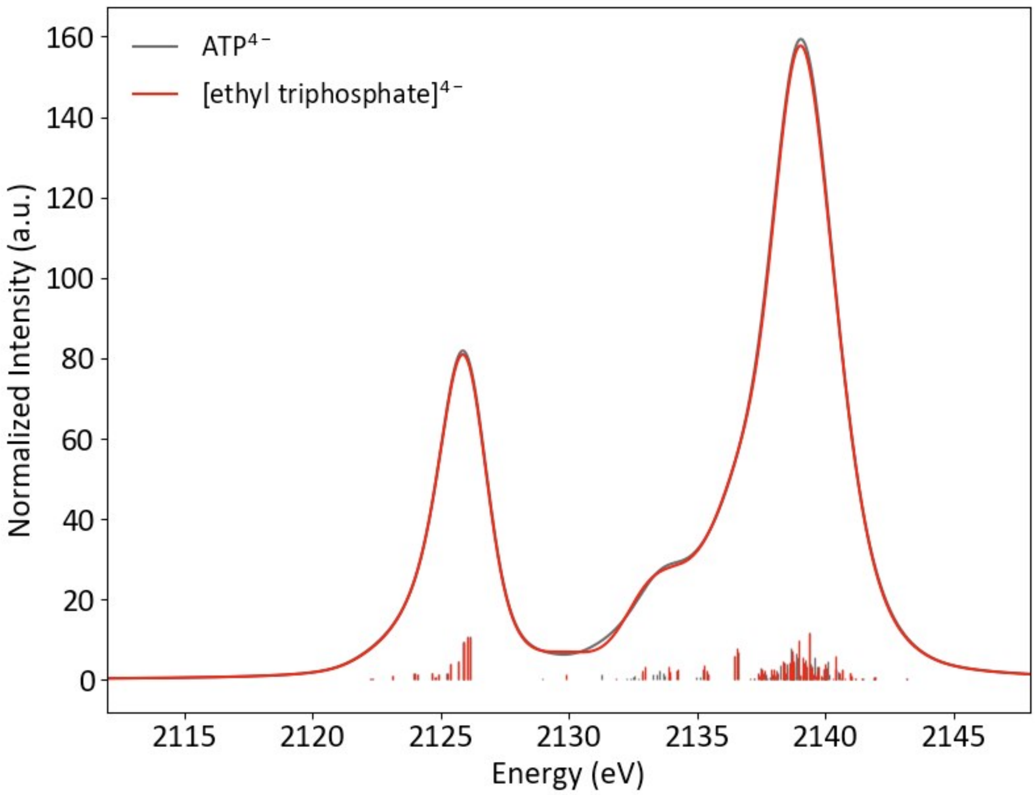

Figure S5. Calculated P Kβ spectra of ATP and ethyl triphosphate

5. MgATP Structures

The conformation of ATP changes drastically when it chelates a dication like Mg^{2+}, with the triphosphate wrapping around the dication (Figure S6).[8] The presence of Mg^{2+} influences the P Kβ spectra of MgATP both via this change in conformation and via its direct stabilization of phosphate-localized MOs (Figure S7).

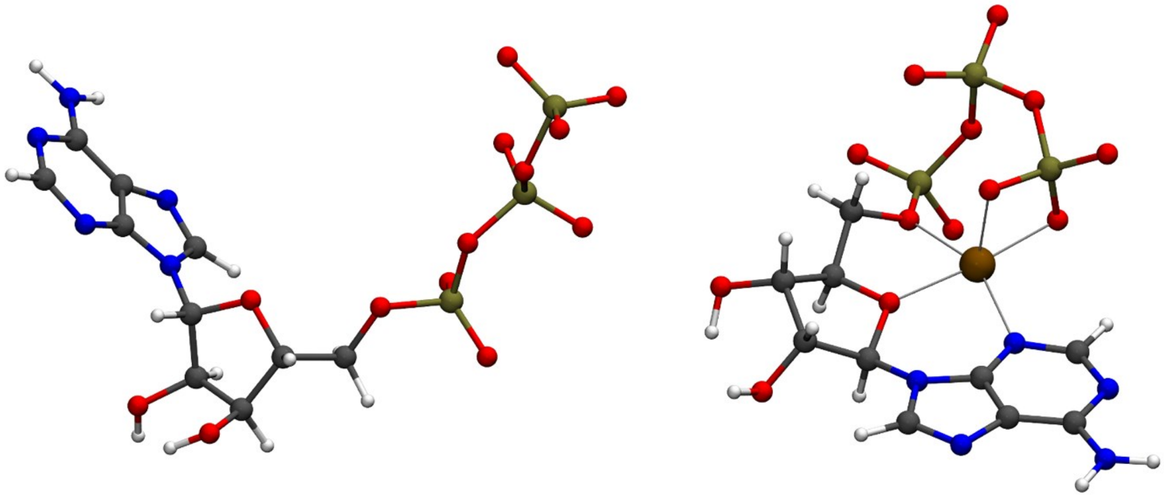

Figure S6. DFT-optimized structures of ATP^{4-} and $MgATP^{2-}$

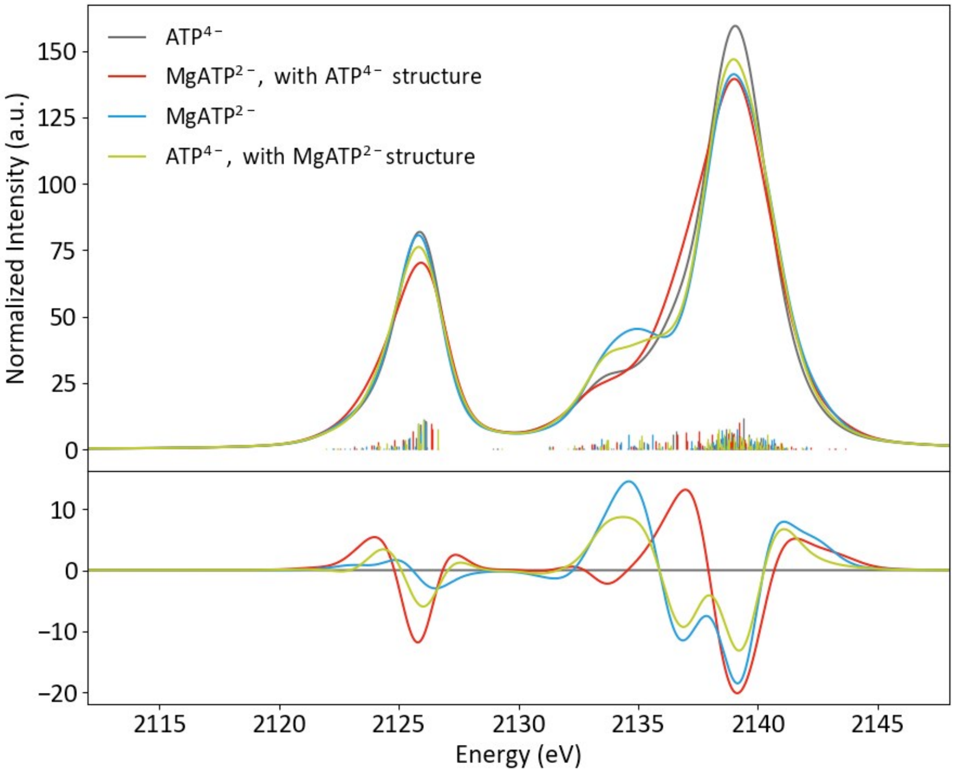

Figure S7. Spectra of ATP^{4-}(optimized), $MgATP^{2-}$ (frozen, with only the Mg^{2+} optimized), $MgATP^{2-}$ (optimized), and ATP^{4-}($MgATP^{2-}$ structure, with Mg^{2+} removed); with differences (x - ATP^{4-} optimized)

6. NADP and NADPH Structures

Continuum-solvated DFT geometry optimizations of NADP and NADPH consistently converged to structures with a hydrogen bond from the nicotinamide to the phosphate with which it shares a ribose (Figure S8).

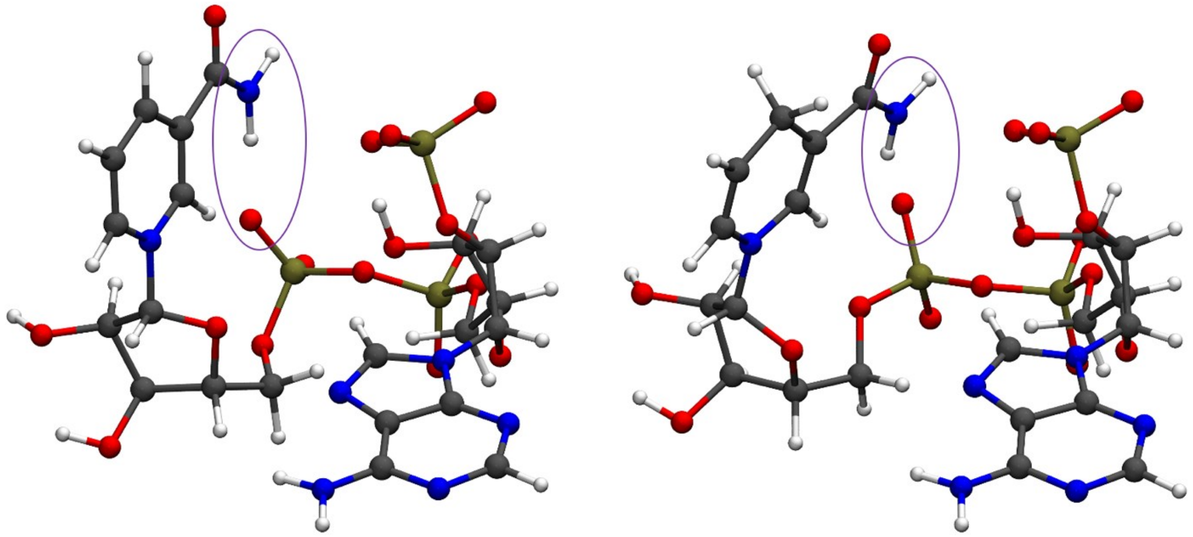

Figure S8. DFT-optimized structures of NADP (left) and NADPH (right), with intramolecular hydrogen bonds from nicotinamide circled

References

1 K. Hamalainen, S. Manninen, P. Suortti, S. P. Collins, M. J. Cooper and D. Laundy, Resonant Raman scattering and inner-shell hole widths in Cu, Zn and Ho, *J. Phys. Condens. Matter*, 1989, **1**, 5955–5964.

2 F. M. F. de Groot, M. H. Krisch and J. Vogel, Spectral sharpening of the Pt L edges by high-resolution x-ray emission, *Phys. Rev. B*, 2002, **66**, 195112.

3 J. L. Campbell and T. Papp, Widths of the Atomic K–N7 Levels, *At. Data Nucl. Data Tables*, 2001, **77**, 1–56.

4 Y. Ito, T. Tochio, M. Yamashita, S. Fukushima, A. M. Vlaicu, Syrocki, K. Słabkowska, E. Weder, M. Polasik, K. Sawicka, P. Indelicato, J. P. Marques, J. M. Sampaio, M. Guerra, J. P. Santos and F. Parente, Structure of high-resolution $K\beta_{1,3}$ x-ray emission spectra for the elements from Ca to Ge, *Phys. Rev. A*, 2018, **97**, 1–10.

5 P. Glatzel and U. Bergmann, High resolution 1s core hole X-ray spectroscopy in 3d transition metal complexes - Electronic and structural information, *Coord. Chem. Rev.*, 2005, **249**, 65–95.

6 H. Enkisch, C. Sternemann, M. Paulus, M. Volmer and W. Schülke, 3d spectator hole satellites of the Cu $K\beta_{1,3}$ and $K\beta_{2,5}$ emission spectrum, *Phys. Rev. A*, 2004, **70**, 022508.

7 T. Mukoyama and K. Taniguchi, Atomic excitation as the result of inner-shell vacancy production, *Phys. Rev. A*, 1987, **36**, 693–698.

8 H. Sigel and R. Griesser, Nucleoside 5′-triphosphates: Self-association, acid-base, and metal ion-binding properties in solution, *Chem. Soc. Rev.*, 2005, **34**, 875–900.
